# Diffuse Leukoplakia of the Bladder Ostium-Sparing in Patient Treated with Leuprorelin for Breast Cancer

**DOI:** 10.1155/2021/9970711

**Published:** 2021-07-27

**Authors:** Antonio Nacchia, Ferdinando di Giacomo, Arcangelo Di Cerbo, Massimo Dante Di Somma, Giuseppe Patitucci, Giuseppe Disabato, Giulia Vita

**Affiliations:** ^1^Urology Department, IRCCS CROB, Rionero in Vulture (PZ), Italy; ^2^Anatomical Pathology Department, IRCCS CROB, Rionero in Vulture (PZ), Italy

## Abstract

**Case:**

A 55-year-old woman came to our attention in April 2020 referring haematuria, frequency and urgency. The patient referred previous treatment with leuprorelin 3.75 mg/2 ml for breast cancer three years ago. Urine culture was performed and resulted always negative for pathogens. Cystoscopy revealed a whitish plaque lesion on the fundus, dome, trigone, and left lateral wall of the bladder. Histology of the biopsy confirmed the diagnosis of leukoplakia of the bladder. The plan is to follow her up repeating a cystoscopy every three months and biopsy in 6 months. Literature search revealed very little information on pathogenesis and prognosis of this condition due to its rare occurrence. The main objective of our case study was to describe individual situation of a woman affected by diffuse leukoplakia of the bladder ostium-sparing with a previous treatment with leuprorelin 3.75 mg/2 ml for breast cancer and to show safety of follow-up by cystoscopy and biopsy.

**Conclusions:**

We showed a case of a woman treated with leuprorelin and with diffuse leukoplakia of the bladder. We support the recommended long-term follow-up and surveillance based on the literature review by cystoscopy with or without biopsy.

## 1. Introduction

Leukoplakia vesicae (LV), also known as keratinizing squamous metaplasia, is a rare histological change of the bladder predisposing the individual to a possible high risk of bladder carcinoma in the background of prolonged exposure to a chronic irritant [1, 2]. It is rarely encountered in urological practice with an incidence of 1 : 10000. In areas where schistosomiasis is uncommon, it usually occurs due to chronic irritation of the inflamed bladder mucosa by bacteria. Risk factors include chronic catheterization, neurogenic bladder, vitamin A deficiency, urinary fistulas, and bladder outlet obstruction [3, 4]. Literature search revealed few reported cases of LV. We are reporting one case of this histological change in the bladder not secondary to chronic irritation due to infection. The main objective of our case study was to describe individual situation of a woman affected by diffuse leukoplakia of the bladder ostium-sparing with previous treatment with leuprorelin 3.75 mg/2 ml for breast cancer and to show safety of follow-up by cystoscopy and biopsy.

## 2. Case Report

**Figure 1:**
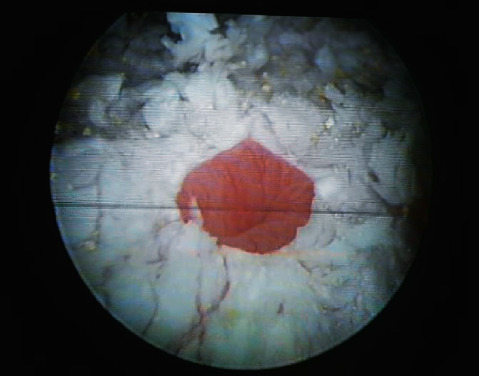
Whitish plaque extended near the left ureteric orifice without interesting it.

**Figure 2:**
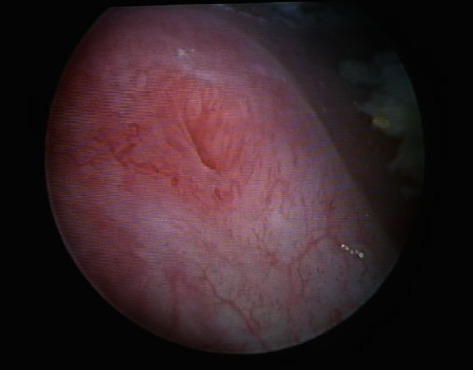
Right orifice was completely spared by plaques.

**Figure 3:**
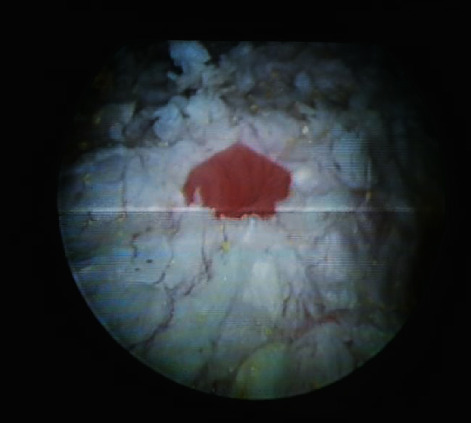
The mucosa underneath the plaques was inflamed.

**Figure 4:**
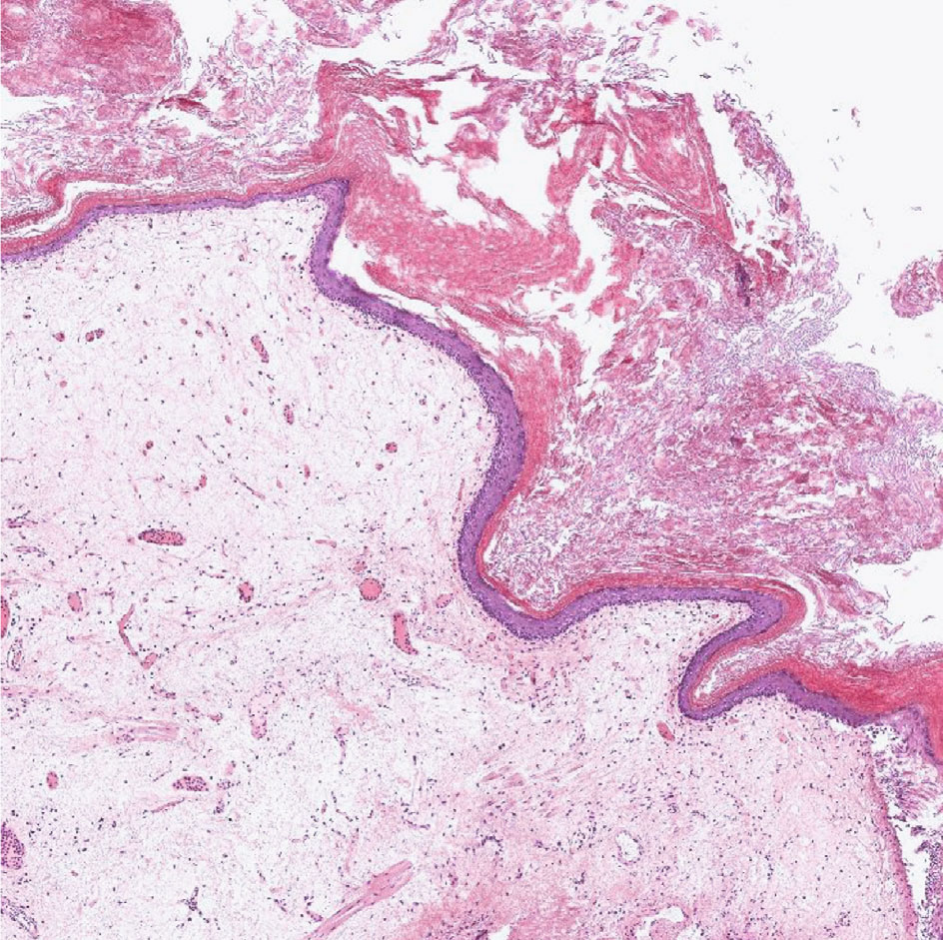
Replacement of the urothelium with stratified keratinized squamous epithelium.

A 55-year-old woman presented gross haematuria for several days. She had history of storage lower urinary tract symptoms (LUTS) since undergoing quadrantectomy surgery for breast cancer 3 years previously. She referred previous treatment with leuprorelin 3.75 mg/2 ml. She was an ex-smoker with a cigarette consumption of maximum ten a day. She interrupted smoking when she was diagnosed breast cancer. She referred past episodes of haematuria and storage LUTS that she always treated with antibiotics even if urine culture was negative. In April 2020, she came to our ambulatory of urology. She showed us a recent urine culture negative for infections and urinary cytology negative for malignant cells. Cystoscopy was performed, and it demonstrated an extensive whitish plaque area on the fundus, the dome, left emi-trigone, and left bladder wall. It extended near the left ureteric orifice without interesting it (Figure 1). Right orifice was completely spared (Figure 2). The efflux from both the ureteric orifices was normal. The mucosa underneath the plaques was inflamed (Figure 3*).* Multiple biopsies were performed. A net margin separated sane mucosa from pathological plagues. The histology of the affected area revealed keratinizing squamous metaplasia and focal low grade epithelial dysplasia (Figure 4). With these collected data, the main objective of our case study was to describe individual situation of a woman affected by diffuse leukoplakia of the bladder ostium-sparing with previous treatment with leuprorelin 3.75 mg/2 ml for breast cancer and to show safety of follow-up by cystoscopy and biopsy.

## 3. Discussion

**Figure 5:**
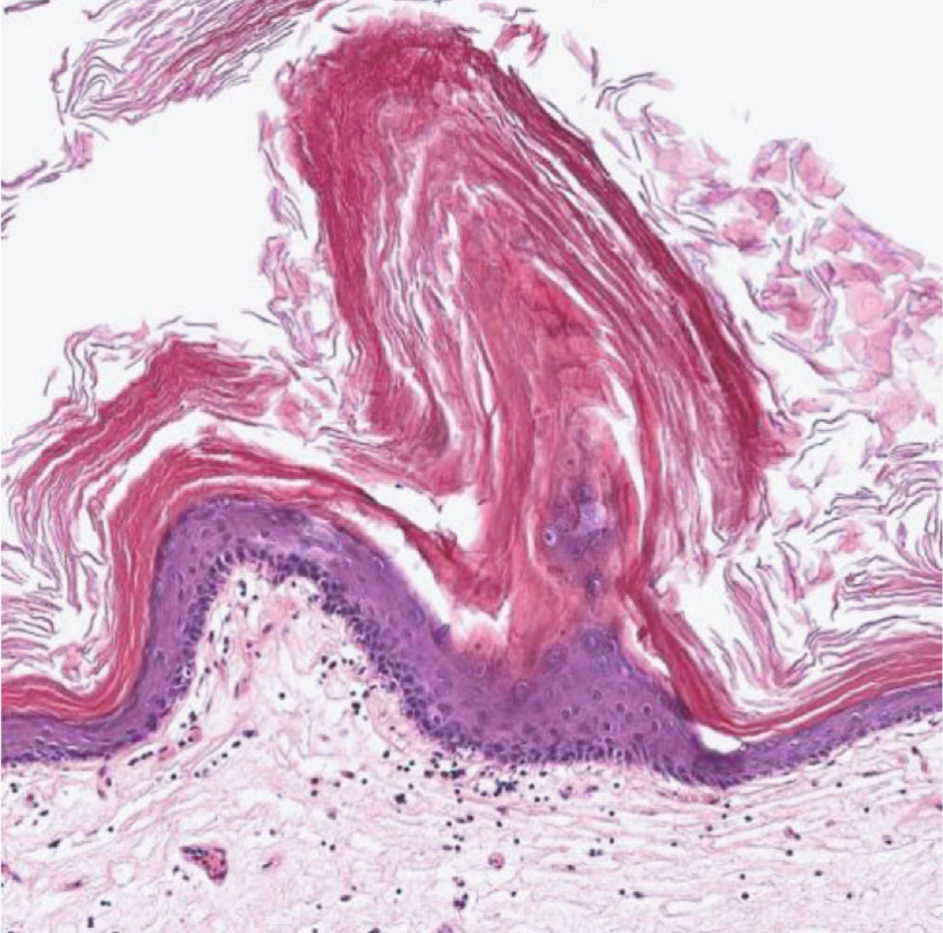
Hyperkeratotic, acanthotic squamous epithelium lining the lumen of the bladder with papillomatosis.

A women of 55 years old came to our attention for LUTS, especially urgency and frequency, associated with haematuria in April 2020. Urine culture was negative. Urinary cytology was negative for malignant cells. Cystoscopy was performed showing a diffuse white plague ostium-sparing. Left orifice was circumferentially spared by LV (Figure 1). Dedicated informed consent for surgery and for this article was given by subject, and her anonymity is totally preserved. Multiple biopsies were performed with a LV diagnosis. According to some evidences in literature [1–3], the patient was managed with medical treatment. Antibiotics, pain killers, and antimuscarinics were given to manage her storage LUTS. She repeated a cystoscopy at six months from first cystoscopy in October 2020, and mapping of the bladder was performed in December 2020 confirming keratinizing squamous metaplasia (Figures 4 and 5).

**Figure 6:**
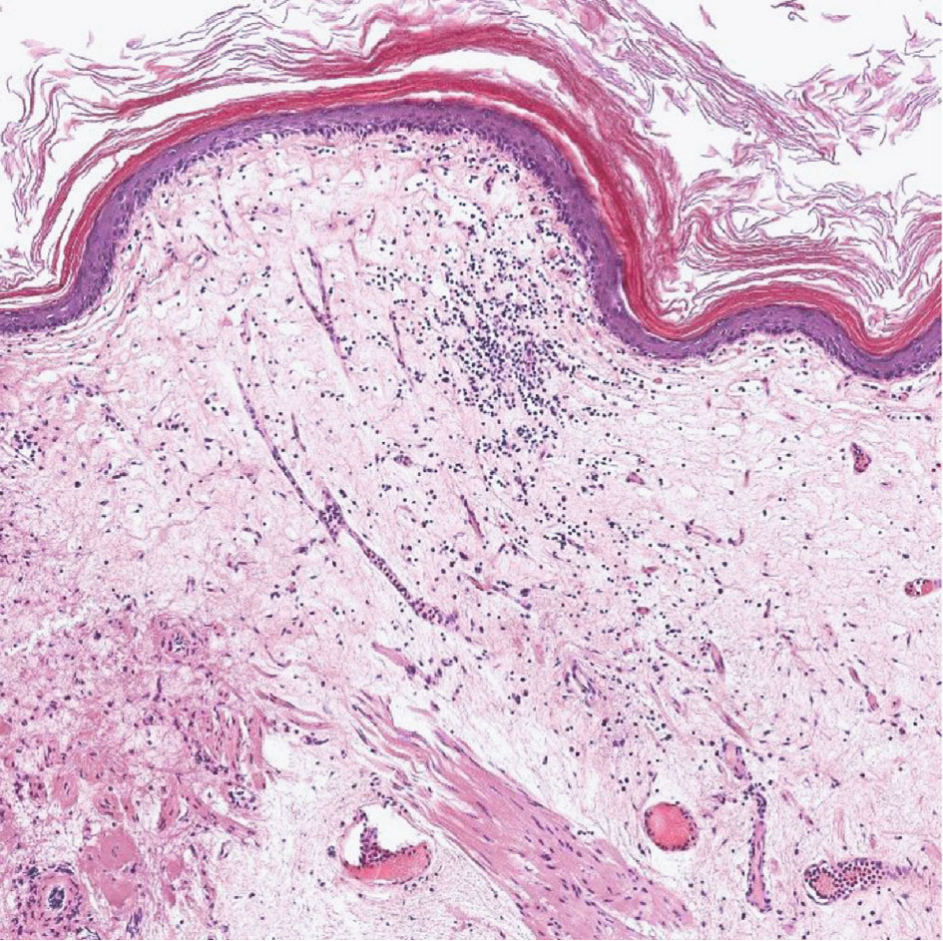
Loose subepithelial connective tissue rich in small vessels with chronic inflammation infiltrate.

Leukoplakia signifies “white plaque”: the pathogenesis being cornification of normally noncornifying membrane due to a chronic inflammation (Figure 6) triggered by various risk factors [5]. It is encountered in urological practice with an incidence of 1 : 10000 and with the highest incidence in women between 50 and 70 years old [1, 5]. Trigone is the commonest site of occurrence sparing the ureteric orifice as in our case, but the condition can also be seen in the other walls of the bladder. Risk factors include chronic catheterization, neurogenic bladder, vitamin A deficiency, urinary fistulas, and bladder outlet obstruction [3].

The etiology is not known exactly, but according to Eyup et al. [2], possible hypotheses are embryological dispersal of ectoderm or spontaneous transformation or secondary epithelial response to appropriate stimuli.

**Figure 7:**
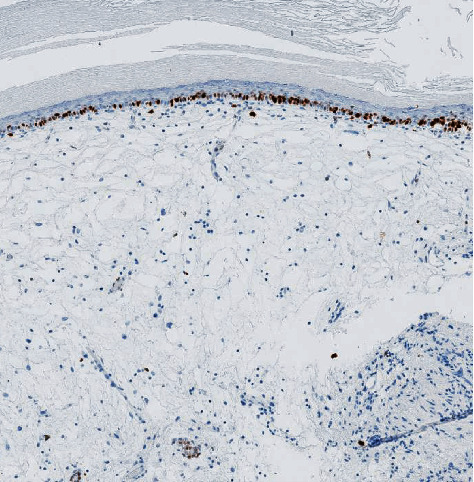
Staining for Ki67 (cell proliferation index) limited to the basal layer of the epithelium supports the benign nature of leukoplakia.

Diagnosis is histological. Microscopically, the normal urothelium is replaced by squamous epithelium with an overlying layer of keratin (Figures 5 and 7). It is considered as a risk factor for squamous cell carcinoma. Based on available literature, the current recommendation is for close cystoscopic monitoring annually to look for any subsequent malignant changes [4].

No consensus on management and treatment is actually available. Antibiotics are the most common therapy used in clinical practice, and they may help symptomatic remission, but efficacy is not durable. Transurethral resection of the bladder therapy significantly relieves urinary symptoms in women with LV. Improvement of quality of life has a success rate of 57.6%. Considering the very low complication rate, our study supports transurethral resection as an alternative treatment for patients who are resistant to medical therapy [6]. Recently, Benelli et al. [7] manage a man affected by LV with hyaluronic acid instillations with resolution of clinical symptoms. It could be considered the starting point and the gold standard in the follow-up of our patient. However, at present, further studies are required to formulate an adequate policy for therapeutic management of this unusual lesion of the bladder mucosa [7].

The woman of this case report was previously treated with leuprorelin 3.75 mg/2 ml for breast cancer. Leuprorelin is in the gonadotropin-releasing hormone (GnRH) analogue family of medications. There are two works evaluating the efficacy of the GnRH-analogue, leuprolide acetate, on NK cell activity. The first one suggests an increased NK cell activity in peripheral blood samples determined by 51Cr release assay [8], and the second one reported that NK cell cytotoxicity from control and patients was significantly decreased with leuprolide acetate [9]. These findings suggest a direct immunomodulatory role of GnRH on NK cell activity.

There is a report where the immunomodulation exerted by GnRH on freshly isolated primary peritoneal macrophages is clearly observed. In this work, the authors found that the production of nitric oxide, costimulated with lipopolysaccharide (LPS) and interferon-*γ* (IFNy), and the activity of NF-*κ*B were suppressed by GnRH exposure. These results demonstrate that GnRH participates in macrophage function and indicate that the NF-*κ*B signaling pathway may be responsible for GnRH-mediated immune modulation [9].

In some studies, leuprorelin was associated with interstitial lung diseases, granulomas, or other kinds of cutaneous eruptions (erythematous macules, infiltrated plaques, subcutaneous nodules, and sterile abscesses) [10–14], but no case reports of LV associated with leuprorelin are reported, so our manuscript should be considered innovative as first article on this topic. Possible association between leuprorelin treatment and LV could be related to alterations on immune system as proposed for interstitial lung disease by Shioi et al. [10]. This possible explanation about immune reactivity was supported for granulomas leuprorelin-related by Yasukawa et al. too [12]. However, etiology and pathogenesis about this association are actually not clear and should be clarified in the future.

## Data Availability

The data that support the findings of this study are available from the corresponding author, A.N., upon reasonable request.

## Additional Points

*Learning Points*. LV is a rare condition which aetiology is not known exactly. Leuprorelin was associated with interstitial lung diseases, granulomas, or other kinds of cutaneous eruptions (erythematous macules, infiltrated plaques, subcutaneous nodules, and sterile abscesses). Leuprorelin use could be associated with LV too through immune system pathways. Recommended long-term follow-up and surveillance by cystoscopy with or without biopsy are safety and should be performed in LV.
